# Supplementary material for: Characterization and Phylogenetic Analysis of MADS-Box Gene Family in Magnoliids: Insights into the Evolution of Floral Morphogenesis in Angiosperms
Source: Plants (Basel). 2025 Sep 27;14(19):2991. doi: 10.3390/plants14192991 (PMC12526163; doi:10.3390/plants14192991)
Supplement: Supplementary file 1 [file plants-14-02991-s001.zip › Plants-S.pdf]

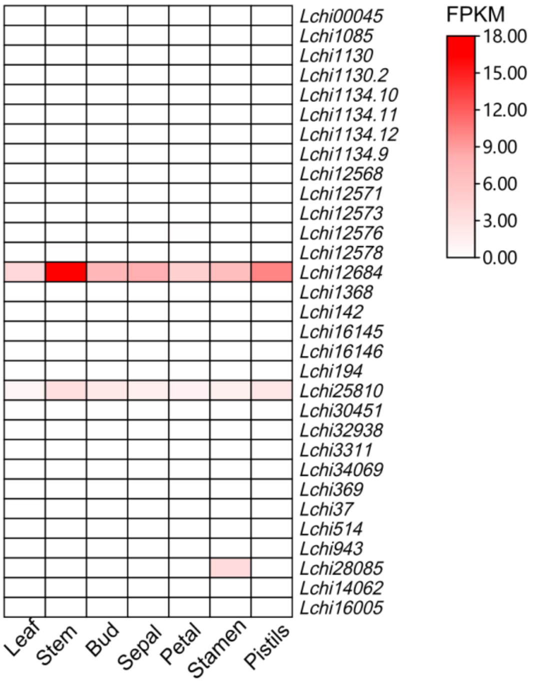

Figure S1. Expression profiles of Type-I MADS-box genes in *Liriodendron chinensis*.

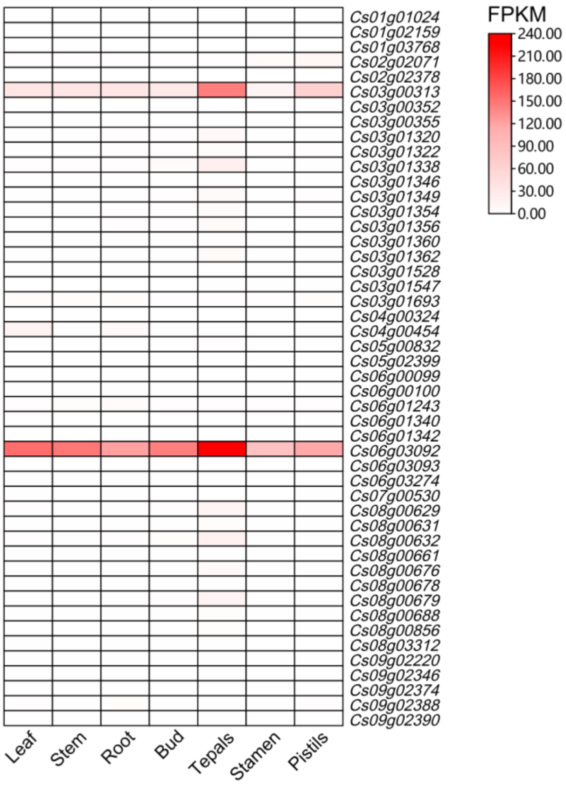

Figure S2. Expression profiles of Type-I MADS-box genes in *Chimonanthus praecox*.
